# Electrophysical Characteristics of Acrylonitrile Butadiene Styrene Composites Filled with Magnetite and Carbon Fiber Fillers

**DOI:** 10.3390/polym16152153

**Published:** 2024-07-29

**Authors:** Elena A. Lebedeva, Elena V. Ivanova, Denis K. Trukhinov, Tatiana S. Istomina, Nikolay S. Knyazev, Alexander I. Malkin, Victor A. Chechetkin, Alexey N. Korotkov, Maria Balasoiu, Svetlana A. Astaf’eva

**Affiliations:** 1 “Institute of Technical Chemistry of UB RAS”—Affiliation of Perm Federal Research Centre of Ural Branch of Russian Academy of Sciences, Akademika Koroleva str., 3, Perm 614013, Russia; itch.elena@mail.ru (E.A.L.); dtruhinov@gmail.com (D.K.T.);; 2 Engineering School of Information Technologies, Telecommunications and Control Systems, Ural Federal University, Mira str., 19, Yekaterinburg 620002, Russiaalexander.malkin@urfu.ru (A.I.M.);; 3 Joint Institute for Nuclear Research, Dubna 141980, Russia; masha.balasoiu@gmail.com; 4 “Horia Hulubei” National Institute of Physics and Nuclear Engineering, 077125 Magurele, Romania; 5 R&D CSMBA, Faculty of Physics, West University of Timișoara, 300223 Timișoara, Romania

**Keywords:** electrodynamics properties, ABS plastic, carbon fiber, magnetite, filler orientation

## Abstract

With the rapid development of wireless communication technologies and the miniaturization trend in the electronics industry, the reduction of electromagnetic interference has become an important issue. To solve this problem, a lot of attention has been focused on polymer composites with combined functional fillers. In this paper, we report a method for creating an acrylonitrile butadiene styrene (ABS) plastic composite with a low amount of conductive carbon and magnetic fillers preparation. Also, we investigate the mechanical, thermophysical, and electrodynamic characteristics of the resulting composites. Increasing the combined filler amount in the ABS composite from 1 to 5 wt % leads to a composite conductivity growth of almost 50 times. It is necessary to underline the temperature decrease of 5 wt % mass loss and, accordingly, the composite heat resistance reduction with an increase in the combined filler from 1 to 5 wt %, while the thermal conductivity remains almost constant. It was established that electrodynamic and physical–mechanical characteristics depend on the agglomeration of fillers. This work is expected to reveal the potential of combining commercially available fillers to construct effective materials with good electromagnetic interference (EMI) protection using mass production methods (extrusion and injection molding).

## 1. Introduction

Advances in wireless communications technology, utilization of wireless communication methods, and miniaturization trends in the electronics industry have increased the importance of reducing electromagnetic interference (EMI) [1]. Electromagnetic radiation impact on the human body and electronics reveals the significance of its minimization [2,3,4,5,6]. To provide a shield against the effects of EMI irradiation, a wide range of different materials are used [7]. The most commonly applied materials are metal screens, which work by reflecting the incident electromagnetic (EM) wave. Magnetic losses in such shielding materials allow for increasing the shielding efficiency in the low-frequency range [8]. The uses of metal screens are limited not only by their characteristics, such as high weight, low corrosion resistance, and manufacturing complexity of complex shape screens, but also by the main shielding mechanism of such materials—the reflection of EM waves, which does not eliminate the impact of interference both inside the screen and through the galvanic connection of the screen to grounding [9].

According to the issues described above inherent to the metal screens, composite shielding materials based on various organic polymer materials are of profound interest [8,9,10]. However, almost all commercially available polymers are good dielectrics. Plastics utilization for screens creation allows for changing the mechanism of EM waves shielding from EM waves reflection to the absorption of energy in the volume of the material through the use of various fillers. The Mao-Sheng Cao research group presented theoretical models for the regulation of dielectric/magnetic behavior, which is important for achieving a balance between reflection and absorption in composite materials [11,12,13,14].

A wide range of synthetic polymers have been studied as dielectric matrices for shielding composites, including polyaniline, polyphenylene sulfide, polyimide, polypropylene (PP), polycarbonate (PC), polyamide, and polylactic acid (PLA) [15]. The primary objective for a shielding material based on a plastic matrix creation is the selection of conductive fillers that can provide the required level of dielectric losses. The most commonly used conductive fillers are carbon materials of various shapes: nanotubes, graphene oxide, and carbon fibers [16,17,18,19,20]. The use of magnetic fillers in composites leads to improved impedance, matching at the sample–air interface, as well as increased EM absorption due to internal magnetic losses [21]. The distribution of conductive and magnetic fillers in the polymer matrix forms electrical and thermal conductive paths, which plays a crucial role in EMI shielding efficiency (SE) and thermal conductivity growth [22]. An increasing amount of literature is devoted to the combined use of nanoscale conductive and magnetic fillers [18,19,20,23,24]. The work [19] shows that the magnetite Fe_3_O_4_ content increase to 10 vol % promotes the development of a percolation network in systems of hybrid PBS nanocomposites with 0.1 vol % of multiwalled carbon nanotubes (MWCNT) and the achievement of high conductivity values. Fang Ren et al. [20] showed a significant conductivity growth of a system with a layered composite structure with 5 wt % of graphene nanosheets (GNS) and 15 wt % of Fe_3_O_4_. Reclaimed carbon fiber (rCF) is not inferior in its properties compared to virgin CF, which makes it an ideal inexpensive and ecofriendly conductive material for EM shielding thermosetting polymer composites reinforcement [25,26,27]. A detailed investigation of the electrodynamic parameters revealed the attractive properties of rCF as a component of shielding materials, especially with the simultaneous addition of magnetite [28].

Plastics and composites based on ABS are used as structural elements of many electronic products and electrical accessories [17]. Mechanical and thermal properties are vital for such devices. Thermal conductivity reduction in ABS plastic-based composite materials can lead to the additional heating of the device and, as a result, to the degradation of its technical and operational characteristics [29].

Our current research is focused on the investigation of the concentration effect of available conductive and magnetic fillers in a widely used ABS matrix to achieve a good correlation between commercial availability and the thermal, mechanical, and electrical properties of the resulting composite material.

## 2. Materials and Methods

### 2.1. Materials

The carbon polyimide is based on epoxy resin ED-20, polyethylenepolyamine (PEPA), and reinforced with carbon cloth Toho Tenax 3 K (Toho Tenax, Wuppertal, Germany). The mass content of CF in the polymer was 26.5 wt %. The ABS plastic (bar diameter 1.75 ± 5 mm) was manufactured by “Hi-Tech Plast” (Vladimir, Russia). All chemical reagents were high purity grade and used as received: iron (II) sulfate heptahydrate (FeSO_4_•7H_2_O) (chemically pure), 12.5 M aqueous ammonia solution (NH_4_OH) (pure for analysis), and iron (III) chloride hexahydrate (FeCl_3_•6H_2_O) (pure) were purchased from “NevaReaktiv” (Saint-Petersburg, Russia); a 95% sulfuric acid (H_2_SO_4_) was purchased from UCC “Shchekinoazot” Ltd. (Tula, Russia); a 37% hydrogen peroxide (H_2_O_2_) and acetone (C_3_H_6_O) (pure for analysis) were purchased from LLC “Sigmatek” (Saint-Petersburg, Russia).

### 2.2. Composite Preparation

The reclaimed carbon fiber was obtained by low-temperature solvolysis [30] with a mixture of H_2_SO_4_/H_2_O_2_/H_2_O_(*dist)*_ (2.5:3:1). According to the scanning electron microscope (SEM) results, the diameter of the rCF was 6–7 μm, and the length of the short carbon fibers was approximately 0.5–2 mm [30]. The magnetic particles were obtained by the addition of ammonium hydroxide excess to the solution of Fe(II) and Fe(III) [28]. The average particle size of Fe_3_O_4_ was 27 nm [28].

**Figure 1:**
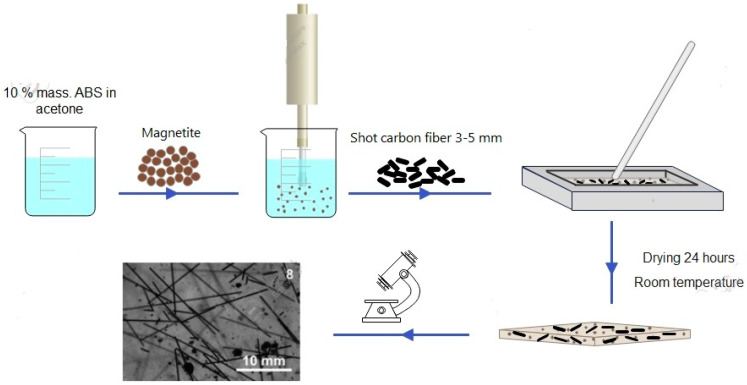
Preparation of ABS samples with functional fillers.

The process of composite preparation based on ABS plastic is shown in the scheme (Figure 1). The preparation of the composite involved dissolving a rod of ABS plastic in acetone to obtain a solution with a 10 wt % plastic. For the initial composite, the resulting solution was poured into a fluoroplastic mold and mechanically mixed to avoid a large number of bubbles in the sample. Mechanical stirring was stopped when the minimum amount of acetone was reached, and the sample was left until the complete evaporation of the acetone at room temperature. The amount of filler and the names of the obtained samples are shown in Table 1.

### 2.3. Characterization

A thermal behavior investigation of the plastics samples was performed using a TGA/DSC 3+ (Mettler Toledo, Greifensee, Switzerland) in the temperature range of 25–1000 °C, with a heating rate of 10 °C/min in an air atmosphere, using 70 μL alumina crucibles as the sample holders. A Differential Scanning Calorimeter (DSC) analysis of the plastics samples was carried out on a DSC1 822e instrument from Mettler Toledo (Greifensee, Switzerland) in an air environment from 0 to 480 °C at a heating rate of 5 °C/min. The thermal conductivity of the samples was studied with the utilization of pure gallium metal and naphthalene. The measurements were performed on a DSC differential scanning calorimeter 882e/400 (Mettler Toledo, Greifensee, Switzerland). The tested material samples had a cylindrical shape, with a diameter of ~5 mm and a thickness of 2–3 mm. The thermal conductivity coefficient was determined in accordance with ASTM E1952-11 [31], and specific heat capacity was determined in accordance with ISO 11357-4:2005 [32]. The test was repeated 3 times, and the error bars were adopted based on the std error calculated from the standard deviation of the mean values.

The physical and mechanical characteristics of the plastics samples were determined on an INSTRON-3365 (High Wycombe, Great Britain) universal testing machine: nominal strength σ_k_; stress–strain modulus E100 at 100%; relative critical tension strain ε_k_, at a tensile speed of 10 mm/min and 25 °C. The samples were thin plates (50 × 10 mm), in accordance with GOST 14236-81 [33]. The thickness of all the samples was 0.25 ± 0.01 mm. The number of strength test repetitions in the series was 4.

After mechanical measurements, the surface of the polymer samples was studied on a FEI Quanta 650FEG scanning electron microscope (SEM) (FEI, Hillsboro, OR, USA) with the following parameters: low vacuum mode, SE, WD ~ 9–7 mm, magnification of 1000 times at 20 kV. 

The distribution of the filler particles in the polymer volume was assessed using an OLIMPUS X501 optical microscope (Tokyo, Japan).

The most important parameters for materials that are used to prevent the propagation of electromagnetic interference are relative permittivity and permeability. To find the frequency dependence of the complex relative permittivity and permeability, we used a measuring equipment based on the vector network analyzer R&SZVA50 (Rohde & Schwarz, Munich, Germany), with the widely used transmission–reflection method, based on existing types of radio frequency (RF) transmission lines. The studied material was placed in a sampler holder with subsequent measurement of the complex coefficients of the scattering matrix. The studied samples were prepared according to waveguide WR90 IEA type in the form of 23 × 10 × 1 mm plates. The complex relative permittivity measurements and the conductivity calculation were made for three samples of each composition material in order to eliminate errors in the preparation of the samples. To obtain the values of complex relative permittivity and permeability, a developed specialized software was used [34]. The calculated error in the real part of the relative permittivity measurement was no more than 5% and is determined by the error of the device itself [35].

## 3. Results and Discussion

### 3.1. Thermal and Mechanical Properties of Samples

In order to study the influence of nature and concentration of introduced fillers (rCF and magnetite) on the thermal properties of the ABS-plastic-based samples, the process of thermal oxidative destruction of the pure ABS plastic and its composites was investigated with a simultaneous thermal analysis (TGA/DSC) method. The thermal oxidative stability comparison of composite samples was carried out according to the temperature corresponding to the 5% weight loss and is based on the thermogravimetric analysis (TGA) results. The introduction of all types of fillers in an amount of 1 wt % and magnetite in an amount of 3 wt % leads to an increase in the heat resistance of ABS samples (Table 2). It is necessary to admit that the joint introduction of carbon fiber and magnetite at 1.5 and 2.5 wt % reduces the thermal stability of samples ABS/rCF/Fe_3_O_4_-3 and ABS/rCF/Fe_3_O_4_-5, respectively. The close contact between goethite (FeOOH) ore with high Fe_2_O_3_ content and carbon promotes a rapid reduction reaction to metallic iron [36,37]. Jalil Vahdati Khaki and co-authors showed that reducing the particle size of a composite mixture of hematite and graphite leads to a decrease in the reduction process temperature and increase in the rate of its occurrence [38].

The oxidation of the polybutadiene segment phase in ABS leads to an exothermic and self-accelerating effect at moderate temperatures [39]. Therefore, the heat resistance reduction in the ABS plastic, with the combined introduction of carbon fiber and magnetite, can pose a potential danger when the final material is dried and extruded during the production process. This effect requires more detailed study and is a subject of our future investigation.

Moreover, we determined the glass transition temperature, thermal conductivity and heat capacity of the samples (Table 2). The introduction of all types of fillers led to the limitation of the polymer chains’ mobility, causing the glass transition temperature to increase [40,41,42]. No noticeable change in the thermal conductivity and heat capacity of the ABS plastic samples with fillers was detected; there is no noticeable change in thermal conductivity and heat capacity for ABS plastic samples with fillers.

**Figure 2:**
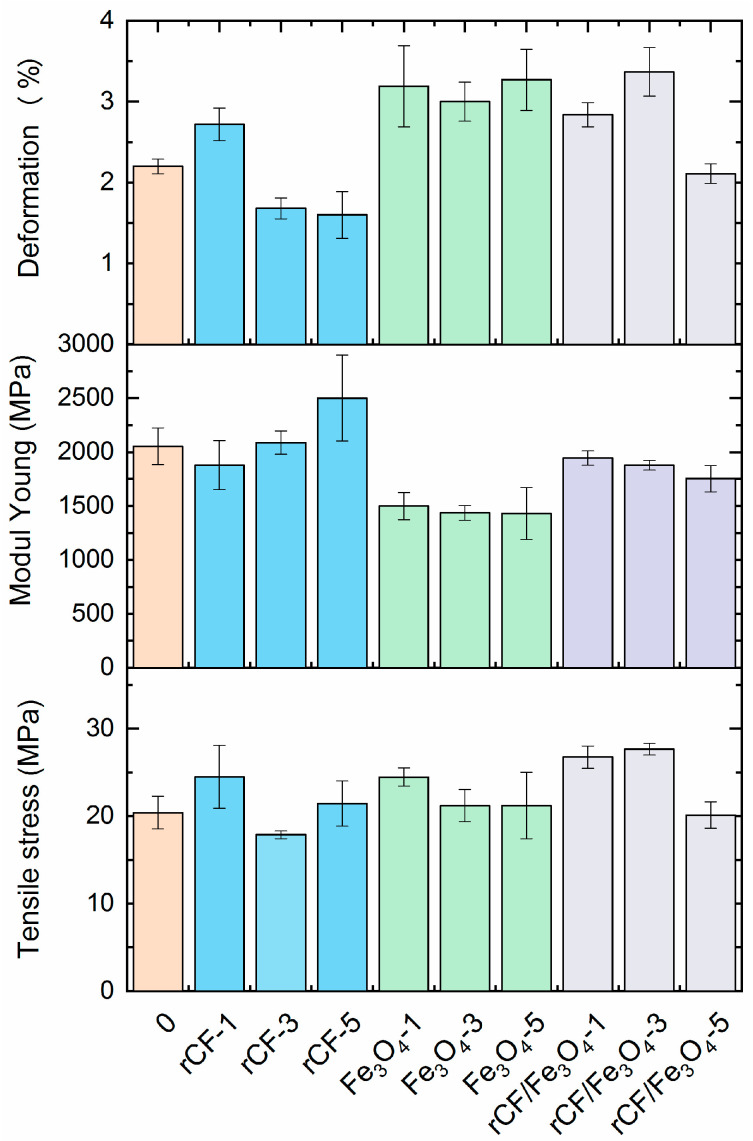
Tensile strength, Young’s modulus and deformation test results for ABS filled with extracted carbon fibers and without filler.

The interaction between the binder and fillers affects the thermal and electrodynamic properties, as well as the strength of the resulting materials [43]. The mechanical properties tests results of the original and filled ABS plastic samples are presented in Figure 2. It is clear that the introduction of the 1 wt % fillers (for ABS/rCF-1, ABS/Fe_3_O_4_-1) and the 3 wt % combined filler (for ABS/Fe_3_O_4_-3) resulted in a mechanical strength growth of more than 20%. In other cases, we observed minor changes in strength. With the addition of the 3 wt % carbon fiber, a strength reduction of 12% was detected, compared to the pure ABS plastic. One of the reasons for the strength decrease in the samples may be the agglomeration of rCF and magnetite particles as their amount increases [44]. In this work, we used a powder filler—magnetite and fiber filler—and a short, reclaimed carbon fiber. Both fillers, in addition to their different adhesion to the polymer matrix, have a difference in form, which can have a significant impact on the agglomeration of the filler during the manufacturing process of the samples and, as a consequence, on the formation of the final characteristics of the material.

**Figure 3:**
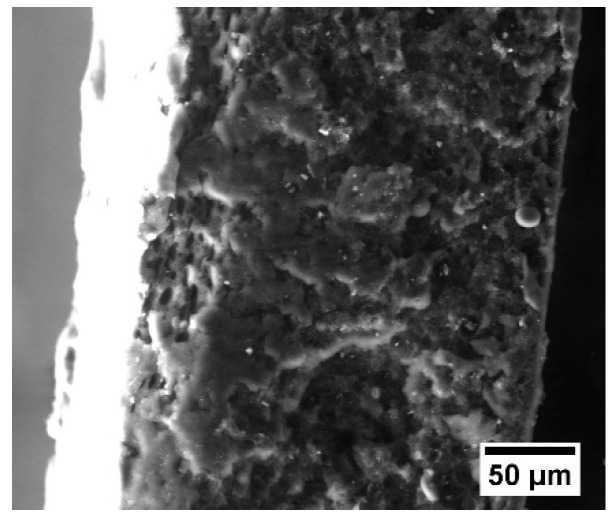
Microphotograph of the ABS plastic sample fracture surface.

**Figure 4:**
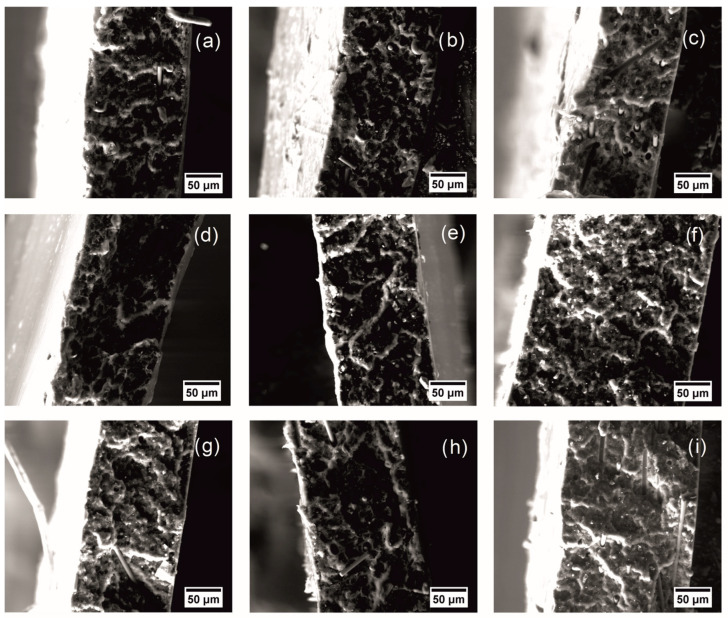
Microphotographs of the ABS plastic samples fracture surfaces filled with rCF 1% (**a**), 3% (**b**), 5% (**c**), magnetite 1% (**d**), 3% (**e**), 5% (**f**) and rCF and magnetite at 0.5% (**g**), 1.5% (**h**), 2.5% (**i**).

For the composites primarily filled with the rCF, the morphology of the fracture surface can provide information characterizing adhesion at the filler matrix interface. High interfacial adhesion is responsible for the production of highly effective composites [45]. The morphological features of the ABS samples’ fractured surfaces are presented on SEM micrographs in Figure 3 and Figure 4. The fractured surface of the filled samples differs slightly from the fractured surface of pure ABS. The spikier surface for ABS samples containing magnetite reflects ductile fracture behavior, compared to samples filled with carbon fibers only [44]. All ABS plastic samples filled with carbon fiber are characterized by adhesive failure and fiber pullout [46]. Moreover, the tensile load was not sufficient to cause fiber failure after matrix failure, causing the fibers to pull out of the matrix during testing [46]. Also, we observed dark rings around the fibers, due to local deformation of the matrix [46].

**Figure 5:**
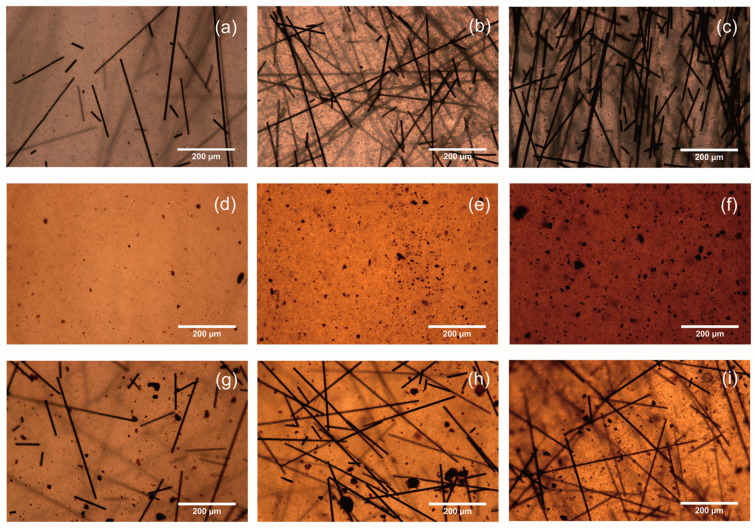
Microphotographs of ABS plastic samples structure filled with rCF 1% (**a**), 3% (**b**), 5% (**c**), magnetite 1% (**d**), 3% (**e**), 5% (**f**) and rCF and magnetite 0.5% (**g**), 1.5% (**h**), 2.5% (**i**).

The ratio of fillers is considered to be one of the main factors that require control and influence the improvement of electrical, thermal, and mechanical properties [47]. In addition, the properties are affected by the distribution of the filler in the polymer matrix and its agglomeration, which can be qualitatively assessed from micrographs of the structure of the samples [48,49]. Figure 5 shows micrographs of the filled ABS plastic samples. For sample ABS/rCF-1 with 1 wt %, the carbon fiber filler is distributed evenly in the ABS matrix, without any continuous structure observed (Figure 5a). Also, a continuous structure is absent for samples with the introduction of magnetite in ABS (Figure 5d–f) and for samples with simultaneous introductions of the 1 wt % carbon fiber (Figure 5g). With the introduction of 3 wt % and 5 wt % carbon fiber, we found areas with high and low carbon fiber content (Figure 5h,i). In this case, we can assume the presence of isolated areas with a conductive structure. It should be noted that the presence of areas with high carbon fiber content also promotes stronger agglomeration of magnetite (Figure 5h,i), compared to the size of the agglomerates (Figure 5e,f).

The fillers’ distribution can also influence the obtained mechanical properties. Thus, a decrease in tensile strength can be noted for compositions with a higher content and greater agglomeration of fillers [22]. A strength improvement of the samples was noted for all samples with 1 wt % of fillers, as well as for the samples with 3 wt % of combined fillers.

### 3.2. Electrodynamic Properties of Composite

The crucial parameters of materials used to ensure compliance with electromagnetic compatibility standards is the electrical conductivity of the dielectric material [21]. One of the main characteristics for composite materials based on a non-conducting dielectric matrix with the addition of carbon fiber is the percolation point. This parameter provides the filler concentrations at which the resulting composite gains sufficient conductivity [50] and, as a consequence, the ability to prevent electromagnetic wave propagation.

Materials that are capable of radiation absorption in the centimeter wavelength range are of great interest for electromagnetic protection. A large number of satellite communication systems, radars, and wireless information transmission systems are implemented in this frequency range, which leads to strong electromagnetic pollution.

The direct measurement of dielectric conductivity as a measure of the material’s ability to conduct electric current at frequencies of several GHz is not correct. At such frequencies, the absorption of electromagnetic energy takes place. To estimate the influence of fillers on these characteristics of the composite, in this work, we used the approach of calculating the real part of the conductivity from the frequency response of the imaginary part of the relative permittivity with the Formula (1) [51]:

σ_d_ = ωε_0_ε_r_″ (1)

where ε_r_″ is imaginary part of relative permittivity, ε_0_ is vacuum permittivity, σ_d_ is conductivity of a dielectric, and ω is angular frequency.

A pure ABS plastic matrix demonstrates stable parameters in the studied frequency range. The measured characteristics are shown in Table 3.

The given characteristics demonstrate that the ABS sample without fillers is characterized by weak conductivity. This indicates that ABS plastic is a radio transparent dielectric material.

**Figure 6:**
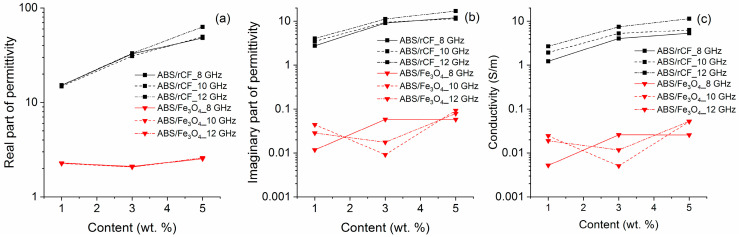
Dependence of real part (**a**) and imaginary part (**b**) of permittivity and conductivity (**c**) on the concentration of fillers.

Figure 6 shows the dependence of the real and imaginary parts of the relative permittivity for the ABS/Fe_3_O_4_ and ABS/rCF compositions on the amount of filler.

According to the dependence, it is clear that the addition of magnetite to the ABS plastic matrix has virtually no effect on the electrodynamic characteristics of the resulting composite. In contrast, the addition of carbon fiber leads to a sharp increase in the value of the real part of the relative permittivity. With the frequency of the applied electromagnetic field’s growth, this dependence becomes steeper (Figure 6a), which may indicate that the electrical length of carbon fibers increases. With the comparison results of the samples structure and the data on their thermal conductivity, heat capacity, and conductivity from Table 2 and Figure 5 and Figure 6, it becomes obvious that the rCF and rCF/Fe_3_O_4_ fillers form an electrically conductive structure [22]. The work [52] shows that larger magnetite particles improve electrical conductivity. Also, larger Fe_3_O_4_ particles increase the conductivity and, therefore, can improve the ability to remove radio frequency (RF) interference for compliance with EMI standards.

The concentration of magnetite filler practically does not affect the imaginary part of the relative permittivity, which can be considered as the imaginary part of a pure ABS plastic matrix. The addition of carbon fiber to ABS plastic matrix sharply increases dielectric losses in the composite. The main reason for such losses is the appearance of conducting regions in the sample, resulting from the addition of conductive carbon fibers. The conductivity dependence on the concentration of fillers is presented in Figure 6c.

**Figure 7:**
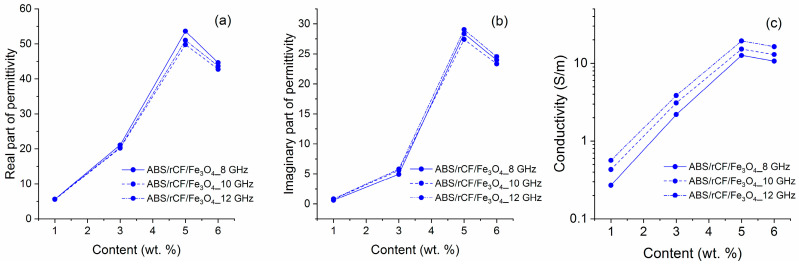
Dependence of the real part (**a**) and imaginary part (**b**) of the relative permittivity and conductivity (**c**) on the concentration of fillers.

Surprisingly, the introduction of Fe_3_O_4_ leads to an increase in conductivity, in contrast to the data given in the work [14]. The simultaneous introduction of magnetite and carbon fiber leads to a more expressed percolation dependence of conductivity, helping to obtain stable electrodynamic characteristics at lower filler concentrations than in the case of addition of carbon fiber only. Figure 7 shows the real and imaginary parts of the relative permittivity dependence of the composite on the filler concentration, rCF/Fe_3_O_4_.

The obtained results allow us to conclude that the addition of magnetite and rCF to the ABS matrix contributes to the production of materials with well-reproducible electrodynamic characteristics, as well as to the achievement of high values of electrical conductivity with a low filler content in the sample.

The data from Table 2 and Figure 6 and Figure 7 suggest a significant difference between the mechanisms of heat and electrical conductivity. We expected that the introduction of carbon fibers and magnetite would not only regulate the electrodynamic characteristics but also increase the thermal conductivity of the samples due to their conductive properties and high aspect ratio [53]. Variation of filler concentration from 1 to 6 wt % for ABS/rCF/Fe_3_O_4_ compositions increased the electrical conductivity without any significant changes in thermal conductivity. Optical microscope images (Figure 5) do not allow for drawing an unambiguous conclusion about the presence of an infinite conducting network in the sample with the maximum amount of fillers. We suppose that carbon fibers and magnetite particles are separated by the layers of a dielectric polymer matrix. Such a structure of composites leads to interfacial resistance between carbon fibers and the polymer matrix, as well as to contact resistance between carbon fibers that have high interfacial thermal resistance, preventing thermal conductivity of samples from increasing. Also, the distribution of fillers in the ABS matrix creates more interfaces that can provide greater phonon scattering than phonon transport, thereby increasing the interfacial thermal resistance and exhibiting low thermal conductivity of the composites [53]. The low thermal conductivity of the samples with rCF and rCF/Fe_3_O_4_ fillers with a significant electrical conductivity growth with increased filler concentration suggests a tunnel or hopping mechanism as the leading mechanism of electrical conductivity. This can also be indirectly confirmed by the conductivity increase with the frequency growth. [15,54,55,56,57,58].

**Figure 8:**
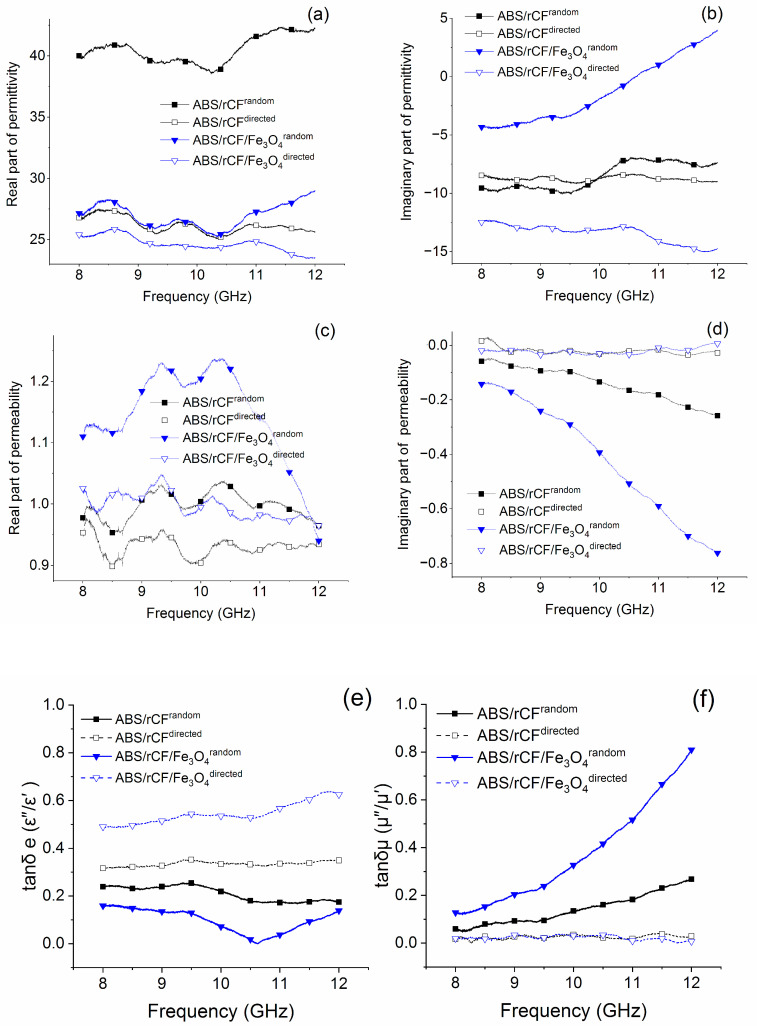
Complex permittivity (**a**,**b**) and permeability (**c**,**d**), tan δ (**e**,**f**) dependence on the orientation of the fiber in the bulk of the material for a composite with the addition of carbon fiber and magnetite.

Since complex permittivity (ε′ and ε″) and complex permeability (μ′ and μ″) are the two most important parameters characterizing the EM response of the materials, we studied the dependence of these characteristics on the frequency of the applied electromagnetic field for composites in case of the direct and random orientation of carbon fibers in the bulk of the polymer. The dependence of the complex permittivity and complex permeability for the mentioned compositions are shown in Figure 8 and indicate that, with different orientations of the fibers, as well as in the presence of magnetite, the main mechanism of electron conduction may change. The maximum values of the real part of the relative permittivity (ε′) are observed for a composite sample ABS/rCF^random^ with a random distribution of carbon fibers. Apparently, this sample is characterized by the maximum number of free electrons [11]. Dielectric losses ε″ do not depend on the relative orientation of CF for samples with carbon fiber only, while for samples with rCF/Fe_3_O_4_ filler, they are significantly higher, with a random distribution of the filler. This feature may indicate that magnetite particles may help to increase the conductivity of the composite.

The analysis of dielectric constant ε′ and ε″ data revealed that, for the random distribution of fibers, the conduction mechanism of electrons predominates for the composition with rCF, while for the composition with rCF/Fe_3_O_4_, the hopping mechanism remains [11].

Figure 8e shows that the tangential loss coefficient tan δ for rCF/Fe_3_O_4_^random^ increases with the frequency growth. With the increasing frequency, losses caused by eddy currents, in conductive composites, increase due to the skin effect leading to the tangential loss coefficient growth [59]. Magnetic losses are also an important factor affecting microwave absorption performance. Due to the skin effect of the conductor and the three-dimensional carbon network, a local conductive network is formed at the macroscopic scale, forming eddy currents and, thus, generating eddy current losses [60]. Figure 8 reveals that μ′ is close to 1 and μ″ is close to 0, indicating weak dynamic magnetic properties arising from weak natural magnetic resonance. The value of tan δ_e_ is higher than tan δ_μ_, which means that dielectric losses are the main way of electromagnetic waves attenuation [59,61,62,63].

The complex permittivity and permeability of compositions with a random distribution of carbon fiber are more dependent on frequency (Figure 8). A detailed study of practical cases on the creation of modern protective materials to ensure compliance with EMI standards contributes to the further development of protective materials.

## 4. Conclusions

In this work, composites containing short, reclaimed CF, magnetite, and their combination were studied. The determination of electrodynamic characteristics showed different conductivity mechanisms in composites with carbon fiber and with a combined carbon fiber/magnetite filler. The introduction of ABS/rCF magnetite leads to a multiple increase in the conductivity of the composition. The thermal properties study of ABS plastics showed that the combined use of carbon fiber and magnetite leads to the thermal stability decrease in the resulting composites, which requires further study. The electrodynamic and physical–mechanical characteristics are dependent on the agglomeration of fillers: a composition with a combined filler of 2.5 wt % carbon fiber and magnetite can be considered optimal. The orientation of a short carbon fiber significantly affects the electrodynamic properties of the composites. To obtain the most stable characteristics, it is necessary to increase the filler orientation in the composites. This results potentially make ABS composites with rCF and magnetite particularly attractive for practical applications as green protective materials using available components.

## Figures and Tables

**Table 1:** Composite samples description.

№	Samples	Amount of Filler, wt %		
		rCF	Fe_3_O_4_	Total Amount
1	ABS/Fe_3_O_4_-1	-	1	1
2	ABS/Fe_3_O_4_-3	-	3	3
3	ABS/Fe_3_O_4_-5	-	5	5
4	ABS/rCF-1	1	-	1
5	ABS/rCF-3	3	-	3
6	ABS/rCF-5	5	-	5
7	ABS/rCF/Fe_3_O_4_-1	0.5	0.5	1
8	ABS/rCF/Fe_3_O_4_-3	1.5	1.5	3
9	ABS/rCF/Fe_3_O_4_-5	2.5	2.5	5
10	ABS/rCF/Fe_3_O_4_-6	3	3	6

**Table 2:** Thermal properties of ABS plastic samples.

Sample	Temperature of the 5% Weight Loss, °C	Glass Transition Temperature, °C	Thermal Conductivity, W/(m•K)	Heat Capacity, kJ/(kg °C)
ABS	345	80.0	0.15	1.36
ABS/rCF-1	354	97.3	0.15	1.30
ABS/rCF-3	349	98.2	0.17	1.28
ABS/rCF-5	346	99.3	0.17	1.25
ABS/Fe_3_O_4_-1	351	89.7	0.15	1.30
ABS/Fe_3_O_4_-3	353	97.8	0.15	1.30
ABS/Fe_3_O_4_-5	346	99.0	0.17	1.30
ABS/rCF/Fe_3_O_4_-1	353	96.2	0.15	1.37
ABS/rCF/Fe_3_O_4_-3	338	97.9	0.15	1.28
ABS/rCF/Fe_3_O_4_-5	338	99.6	0.15	1.23

**Table 3:** The electrodynamic characteristics of a pure matrix of ABS at fixed frequencies.

Frequency, GHz	ε_r_′	ε_r_″	σ_d_, S/m
8	2.4806	0.085	0.037
10	2.4804	0.115	0.064
12	2.4709	0.089	0.059

## Data Availability

The original contributions presented in the study are included in the article, further inquiries can be directed to the corresponding author/s.
